# What is the McGurk effect?

**DOI:** 10.3389/fpsyg.2014.00725

**Published:** 2014-07-10

**Authors:** Kaisa Tiippana

**Affiliations:** Division of Cognitive Psychology and Neuropsychology, Institute of Behavioural Sciences, University of HelsinkiHelsinki, Finland

**Keywords:** audiovisual, illusion, integration, McGurk effect, multisensory, perception, speech

McGurk and MacDonald (1976) reported a powerful multisensory illusion occurring with audiovisual speech. They recorded a voice articulating a consonant and dubbed it with a face articulating another consonant. Even though the acoustic speech signal was well recognized alone, it was heard as another consonant after dubbing with incongruent visual speech. The illusion has been termed the McGurk effect. It has been replicated many times, and it has sparked an abundance of research. The reason for the great impact is that this is a striking demonstration of multisensory integration. It shows that auditory and visual information is merged into a unified, integrated percept. It is a very useful research tool since the strength of the McGurk effect can be taken to reflect the strength of audiovisual integration.

Here I shall make two main claims regarding the definition and interpretation of the McGurk effect since they bear relevance to its use as a measure of multisensory integration. First, the McGurk effect should be defined as a categorical change in auditory perception induced by incongruent visual speech, resulting in a single percept of hearing something other than what the voice is saying. Second, when interpreting the McGurk effect, it is crucial to take into account the perception of the unisensory acoustic and visual stimulus components.

There are many variants of the McGurk effect (McGurk and MacDonald, 1976; MacDonald and McGurk, 1978)^1^. The best-known case is when dubbing a voice saying [b] onto a face articulating [g] results in hearing [d]. This is called the fusion effect since the percept differs from the acoustic and visual components. Many researchers have defined the McGurk effect exclusively as the fusion effect because here integration results in the perception of a third consonant, obviously merging information from audition and vision (van Wassenhove et al., 2007; Keil et al., 2012; Setti et al., 2013). This definition ignores the fact that other incongruent audiovisual stimuli produce different types of percepts. For example, a reverse combination of these consonants, A[g]V[b], is heard as [bg], i.e., the visual and auditory components one after the other. There are other pairings, which result in hearing according to the visual component, e.g., acoustic [b] presented with visual [d] is heard as [d]. Here my first claim is that the definition of the McGurk effect should be that an acoustic utterance is heard as another utterance when presented with discrepant visual articulation. This definition includes all variants of the illusion, and it has been used by MacDonald and McGurk (1978) themselves, as well as by several others (e.g., Rosenblum and Saldaña, 1996; Brancazio et al., 2003). The different variants of the McGurk effect represent the outcome of audiovisual integration. When integration takes place, it results in a unified percept, without access to the individual components that contributed to the percept. Thus, when the McGurk effect occurs, the observer has the subjective experience of hearing a certain utterance, even though another utterance is presented acoustically.

One challenge with this interpretation of the McGurk effect is that it is impossible to be certain that the responses the observer gives correspond to the actual percepts. The real McGurk effect arises due to multisensory integration, resulting in an altered auditory percept. However, if integration does not occur, the observer can perceive the components separately and may choose to respond either according to what he heard or according to what he saw. This is one reason why the fusion effect is so attractive: If the observer reports a percept that differs from both stimulus components, he does not seem to rely on either modality alone, but instead really fuse the information from both. However, this approach does not guarantee a straightforward measure of integration any more than the other variants of the illusion, as is argued below.

The second main claim here is that the perception of the acoustic and visual stimulus components has to be taken into account when interpreting the McGurk effect. This issue has been elaborated previously in the extensive work by Massaro and colleagues (Massaro, 1998) and others (Sekiyama and Tohkura, 1991; Green and Norrix, 1997; Jiang and Bernstein, 2011). It is important because the identification accuracy of unisensory components is reflected into audiovisual speech perception.

In general, the strength of the McGurk effect is taken to increase when the proportion of responses according to the acoustic component decreases and/or when the proportion of fusion responses increases. That is, the McGurk effect for stimulus A[b]V[g] is considered stronger when fewer B responses and/or more D responses are given. This is often an adequate way to measure the strength of the McGurk effect—if one keeps in mind that it implicitly assumes that perception of the acoustic and visual components is accurate (or at least constant across conditions that are compared). However, it can lead to erroneous conclusions if this assumption does not hold.

The fusion effect provides a prime example of this caveat. It has been interpreted to mean that acoustic and visual information is integrated to produce a novel, intermediate percept. For example, when A[b]V[g] is heard as [d], the percept is thought to emerge due to fusion of the features (for the place of articulation) provided via audition (bilabial) and vision (velar), so that a different, intermediate consonant (alveolar) is perceived (van Wassenhove, 2013). However, already McGurk and MacDonald (1976) themselves wrote that “lip movements for [ga] are frequently misread as [da],” even though they did not measure speechreading performance, unfortunately. The omission of the unisensory visual condition in the original study is one factor that has contributed to the strong status of the fusion effect as the only real McGurk effect, reflecting true integration. Still, if visual [g] is confused with [d], it is not at all surprising or special if A[b]V[g] is perceived as [d].

To demonstrate the contribution of the unisensory components more explicitly, I'll take two examples of my research, in which fusion-type stimuli produced different percepts depending on the clarity of the visual component. In one study, a McGurk stimulus A[epe]V[eke] was mainly heard as a fusion [ete] (Tiippana et al., 2004). This reflected the fact that in a visual-only identification task, the visual [eke] was confused with [ete] (42% K responses and 45% T responses to visual [eke]). In another study, a McGurk stimulus A[apa]V[aka] was mainly heard as [aka], and this could be traced back to the fact that in a visual-only identification task, the visual [aka] was clearly distinguishable from [ata], and thus recognized very accurately (100% correct in typical adults; Saalasti et al., 2012; but note the deviant behavior of individuals with Asperger syndrome). Thus, even though the McGurk stimuli were of a fusion type in both studies, their perception differed depending largely on the clarity of the visual components. These findings underscore the importance of knowing the perceptual qualities of the unisensory stimuli before making conclusions about multisensory integration.

Exactly how to take the properties of the unisensory components into account in multisensory perception of speech is beyond this paper. Addressing this issue in detail requires carefully designed experimental studies (Bertelson et al., 2003; Alsius et al., 2005), computational modeling (Massaro, 1998; Schwartz, 2010), and investigation of the underlying brain mechanisms (Sams et al., 1991; Skipper et al., 2007). However, the main guideline is that unisensory perception of stimulus components is reflected into multisensory perception of the whole (Ernst and Bülthoff, 2004).

During experiments, when the task is to report what was heard, the observer reports the conscious auditory percept evoked by the audiovisual stimulus. If there is no multisensory integration or interaction, the percept is identical for the audiovisual stimulus and the auditory component presented alone. If there is audiovisual integration, the conscious auditory percept changes. To which extent visual input influences the percept depends on how coherent and reliable information each modality provides. Coherent information is integrated and weighted e.g., according to the reliability of each modality, which is reflected in unisensory discriminability.

This perceptual process is the same for audiovisual speech—be it natural, congruent audiovisual speech or artificial, incongruent McGurk speech stimuli. The outcome is the conscious auditory percept. Depending on the relative weighting of audition and vision, the outcome for McGurk stimuli can range from hearing according to the acoustic component (when audition is more reliable than vision) to fusion and combination percepts (when both modalities are informative to some extent) to hearing according to the visual component (when vision is more reliable than audition). Congruent audiovisual speech is treated no differently, showing visual influence when the auditory reliability decreases. The different variants of the McGurk effect are all results of this same perceptual process and reflect audiovisual integration.

The McGurk effect is an excellent tool to investigate multisensory integration in speech perception. The main messages of this opinion paper are, first, that the McGurk effect should be defined as a change in auditory perception due to incongruent visual speech, so that observers hear another speech sound than what the voice uttered, and second, that the perceptual properties of the acoustic and visual stimulus components should be taken into account when interpreting the McGurk effect as reflecting integration.

## Conflict of interest statement

The author declares that the research was conducted in the absence of any commercial or financial relationships that could be construed as a potential conflict of interest.

## Footnotes

^1^Throughout this paper only some representative references are mentioned as examples of the extensive literature on each topic.
